# Supplementary material for: MetaNorm: incorporating meta-analytic priors into normalization of NanoString nCounter data
Source: Bioinformatics. 2024 Jan 17;40(1):btae024. doi: 10.1093/bioinformatics/btae024 (PMC10826904; doi:10.1093/bioinformatics/btae024)
Supplement: btae024_Supplementary_Data [file btae024_supplementary_data.pdf]

Supplementary Material

S1 Meta-Analysis Details

Full Probability Model

$$P(\boldsymbol{Y},\Theta) \propto \prod_{k=1}^{K}\left(\prod_{i=1}^{n_k}\left(\prod_{j=1}^{6} N\left(Y_{ijk}|a_{ik}+b_{ik}X_j^{+}+s_{jk},\sigma_{jk}^2\right)\right)\right)\cdot\prod_{k=1}^{K}\prod_{i=1}^{n_k}\left(\mathcal{N}\left(a_{ik},b_{ik}\middle|\begin{pmatrix}\alpha_k\\ \beta_k\end{pmatrix},\Sigma^{(k)}\right)\right)$$

$$\cdot\prod_{k=1}^{K}\left(N\left(\alpha_k\,|\mu_\alpha,\sigma_\alpha^2\right)\cdot N\left(\beta_k\,|\mu_\beta,\sigma_\beta^2\right)\cdot IW_3\left(\Sigma^{(k)}\,|I_2\right)\right)\cdot\pi(\mu_\alpha)\cdot\pi(\mu_\beta)\cdot\pi(\sigma_\alpha^2)\cdot\pi(\sigma_\beta^2)$$

$$\cdot\prod_{k=1}^{K}\prod_{j=1}^{6}\left(N\left(s_{jk}\,|t_j,\tau^2\right)\cdot\pi(\sigma_{jk}^2)\right)\cdot\prod_{j=1}^{6}\left(\pi\left(t_j\right)\right)\cdot\pi(\tau^2)$$

Full Conditional Distributions

Let $\boldsymbol{\theta_{ik}}=\begin{pmatrix}a_{ik}\\ b_{ik}\end{pmatrix}$, $\boldsymbol{X_{ik}}=\begin{bmatrix}1 & X_{i1k}\\ ... & ...\\ 1 & X_{i6k}\end{bmatrix}$, $\boldsymbol{m_k}=\begin{pmatrix}\alpha_k\\ \beta_k\end{pmatrix}$, while $\boldsymbol{Y_{ik}},\boldsymbol{s_k}$ are vectors over all 6 Y_{ijk}, s_{jk} and $\text{diag}(\sigma_{jk}^2)$ is a diagonal matrix with $\sigma_{1k}^2 ... \sigma_{6k}^2$ in the diagonal. $I_{(L,U)}$ is the indicator function, where $I_{(L,U)}=1$ when $L<x<U$ and 0 otherwise. For all Inverse-Gamma (IG) distributions, $\epsilon=.01$.

$$\boldsymbol{\theta_{ik}}|\cdots,\boldsymbol{Y}\sim N\left(\mu_{\alpha\beta},\boldsymbol{\Sigma}_{\alpha\beta}^{(k)}\right) \text{ where}$$

$$\mu_{\alpha\beta}=\boldsymbol{\Sigma}_{\alpha\beta}^{(k)}\left(\boldsymbol{\Sigma}^{(k)^{-1}}\boldsymbol{m_k}+\boldsymbol{X_{ik}^T}\text{diag}(\sigma_{jk}^2)^{-1}(\boldsymbol{Y_{ik}}-\boldsymbol{s_k})\right) \text{ and}$$

$$\boldsymbol{\Sigma}_{\alpha\beta}^{(k)}=\left(\boldsymbol{\Sigma}^{(k)^{-1}}+\boldsymbol{X_{ik}^T}\text{diag}(\sigma_{jk}^2)^{-1}\boldsymbol{X_{ik}}\right)^{-1}$$

$$\boldsymbol{\Sigma}^{(k)}\Big|\cdots,\boldsymbol{Y}\sim IW_{n_k+3}\left(I+\sum_{i=1}^{n_k}\begin{bmatrix}(a_{ik}-\alpha_k)^2 & (a_{ik}-\alpha_k)(b_{ik}-\beta_k)\\ (a_{ik}-\alpha_k)(b_{ik}-\beta_k) & (b_{ik}-\beta_k)^2\end{bmatrix}\right)$$

$$\boldsymbol{m}_k|\cdots,\boldsymbol{Y}\sim BVN\left(\left(\boldsymbol{\Sigma}_m^{-1}+n_k\boldsymbol{\Sigma}^{(k)^{-1}}\right)^{-1}\left(\boldsymbol{\Sigma}_m^{-1}\boldsymbol{\mu}+\sum_{i=1}^{n_k}\left(\boldsymbol{\Sigma}^{(k)^{-1}}\boldsymbol{\theta}_{ik}\right)\right),\left(\boldsymbol{\Sigma}_m^{-1}+n_k\boldsymbol{\Sigma}^{(k)^{-1}}\right)^{-1}\right)$$

$$\mu_\alpha|\cdots,\boldsymbol{Y})\sim I_{(L_\alpha,U_\alpha)}\cdot N\left(\frac{1}{K}\sum_{k=1}^{K}\alpha_k,\frac{\sigma_\alpha^2}{K}\right)$$

$$\mu_\beta|\cdots,\boldsymbol{Y})\sim I_{(L_\beta,U_\beta)}\cdot N\left(\frac{1}{K}\sum_{k=1}^{K}\beta_k,\frac{\sigma_\beta^2}{K}\right)$$

$$\sigma_\alpha^2|\cdots,\boldsymbol{Y}\sim IG\left(\epsilon+\frac{K}{2},\epsilon+\frac{1}{2}\sum_{k=1}^{K}(\alpha_k-\mu_\alpha)^2\right)$$

$$\sigma_\beta^2|\cdots,\boldsymbol{Y}\sim IG\left(\epsilon+\frac{K}{2},\epsilon+\frac{1}{2}\sum_{k=1}^{K}(\beta_k-\mu_\beta)^2\right)$$

For s_{jk}, we apply two constraints and update the parameter vectors s_{5k} and s_{6k} based on $s_{1k}, s_{2k}, s_{3k}, s_{4k}$. Let $h_j = \frac{X_6 - X_j}{X_5 - X_6}$, $r_{ijk} = Y_{ijk} - a_{ik} - b_{ik} X_j$, $S_{1:4,-j} = \left(\sum_{l=1}^{4} s_{lk}\right) - s_{jk}$, $(XS)_{1:4,-j} = \left(\sum_{l=1}^{4} X_l s_{lk}\right) - X_j s_{jk}$.

$$s_{jk} | \cdots, \boldsymbol{Y} \sim N\left(\mu_{jk}^{(s)}, \sigma_{jk}^{2(s)}\right) \text{ where } j = 1, 2, 3, 4$$

$$\sigma_{jk}^{2(s)} = \left(\frac{1}{\tau^2}\left(1 + h_j^2 + (1 + h_j)^2\right) + \frac{n_k}{\sigma_{jk}^2} + \frac{n_k}{\sigma_{5k}^2} h_j^2 + \frac{n_k}{\sigma_{6k}^2}(1 + h_j)^2\right)^{-1}$$

$$\begin{aligned}
\mu_{jk}^{(s)} = \sigma_{jk}^{2(s)} & \left(\frac{t_j}{\tau^2} + \frac{(t_5 - \frac{X_6 S_{1:4,-j} - (XS)_{1:4,-j}}{X_5 - X_6})}{\tau^2} h_j - \frac{t_6 + S_{1:4,-j} + \frac{X_6 S_{1:4,-j} - (XS)_{1:4,-j}}{X_5 - X_6}}{\tau_6^2}(1 + h_j)\right) \\
& + \sigma_{jk}^{2(s)} \left(+\frac{1}{\sigma_{jk}^2} \sum_{i=1}^{n_k} r_{ijk} + \frac{1}{\sigma_{5k}^2} \sum_{i=1}^{n_k} \left(r_{i5k} - \frac{X_6 S_{1:4,-j} - (XS)_{1:4,-j}}{X_5 - X_6}\right) h_j\right) \\
& - \sigma_{jk}^{2(s)} \left(\frac{1}{\sigma_{6k}^2} \sum_{i=1}^{n_k} \left(r_{i6k} + S_{1:4,-j} + \frac{X_6 S_{1:4,-j} - (XS)_{1:4,-j}}{X_5 - X_6}\right)(1 + h_j)\right)
\end{aligned}$$

$$s_{5k} = \frac{X_6 \sum_{j=1}^{4} s_{jk} - \sum_{j=1}^{4} X_j s_{jk}}{X_5 - X_6}, \qquad s_{6k} = -\left(\sum_{j=1}^{5} s_{jk}\right)$$

For t_j, we apply two constraints and update the parameters t_5 and t_6 based on t_1, t_2, t_3, t_4. Note that $T_{1:4,-j}$ and $(XT)_{1:4,-j}$ are defined similarly to $S_{1:4,-j}$ and $(XS)_{1:4,-j}$ above.

$$t_j | \cdots, \boldsymbol{Y} \sim I_{(L_t, U_t)} \cdot N\left(\mu_j^{(t)}, \sigma_j^{2(t)}\right) \text{ where } t = 1, 2, 3, 4$$

$$\sigma_j^{2(t)} = \left(\frac{K}{\tau^2}\left(1 + h_j^2 + (1 + h_j)^2\right)\right)^{-1}$$

$$\mu_j^{(t)} = \sigma_j^{2(t)} \left[\frac{1}{\tau^2}\left(\sum_{k=1}^{K} s_{jk} + h_j \sum_{k=1}^{K}\left(s_{5k} - \frac{X_6 T_{1:4,-j} - (XT)_{1:4,-j}}{X_5 - X_6}\right) - (1 + h_j)\sum_{k=1}^{K}\left(s_{6k} + T_{1:4,-j} + \frac{X_6 T_{1:4,-j} - (XT)_{1:4,-j}}{X_5 - X_6}\right)\right)\right]$$

$$\tau^2 \Big| \cdots, \boldsymbol{Y} \sim IG\left(\epsilon + 3K, \epsilon + \frac{1}{2}\sum_{k=1}^{K}\sum_{j=1}^{6}(s_{jk} - t_j)^2\right)$$

$$\sigma_{jk}^2 | \cdots, \boldsymbol{Y} \sim IG\left(\epsilon + \frac{n_k}{2}, \epsilon + \frac{1}{2}\sum_{i=1}^{n_k}(Y_{ijk} - (a_{ik} + b_{ik} X_{ijk} + s_{jk}))^2\right)$$

S2 Tables and Figures for MetaNorm

Table S1: Meta-Analysis Datasets

ID	Description of samples	# of samples	Reference	GSE ID or Link
1	Lung adenocarcinoma (LUAD) patients	162	Molania et al. (2019)	N/A[1]
2	Inflammatory bowel disease patients	989	Molania et al. (2019)	GSE73094
3	Colon cancer patients	96	Chen et al. (2016)	GSE62932
4	Lung cancer patients	28	Jia et al. (2019)	N/A[2]
5	Colorectal cancer (CRC) patients	54	Jia et al. (2019)	GSE86561
6	Early stage CRC patient tumors	144	Low et al. (2017)	GSE81983
7	Early stage CRC patient tumors	131	Low et al. (2017)	GSE81985
8	Breast tumor samples	1321	Liu et al. (2016)	GSE74821
9	Patients stimulated with anti-CD3/CD28	1950	Molania et al. (2019)	GSE60341
10	Healthy individuals (stimulated and controls)	2441	Molania et al. (2019)	GSE53165
11	Carolina breast cancer study (CBCS) tumors	1278	Patel et al. (2022)	GSE148418
12	Survivors of triple negative breast cancer	254	Cascione et al. (2013)	GSE45498
13	Merkel-cell carcinoma patients	8	Gravemeyer et al. (2021)	GSE159662
14	T-cell lymphoma or dermititis (plus controls)	128	Nielsen et al. (2019)	GSE143382
15	Metastatic melanoma patients	24	DeVito et al. (2021)	GSE165745
16	Breast cancer patients	1253	Pu et al. (2019)	GSE147126
17	Squamous cell carcinoma patients	67	Meehan et al. (2020)	GSE148944

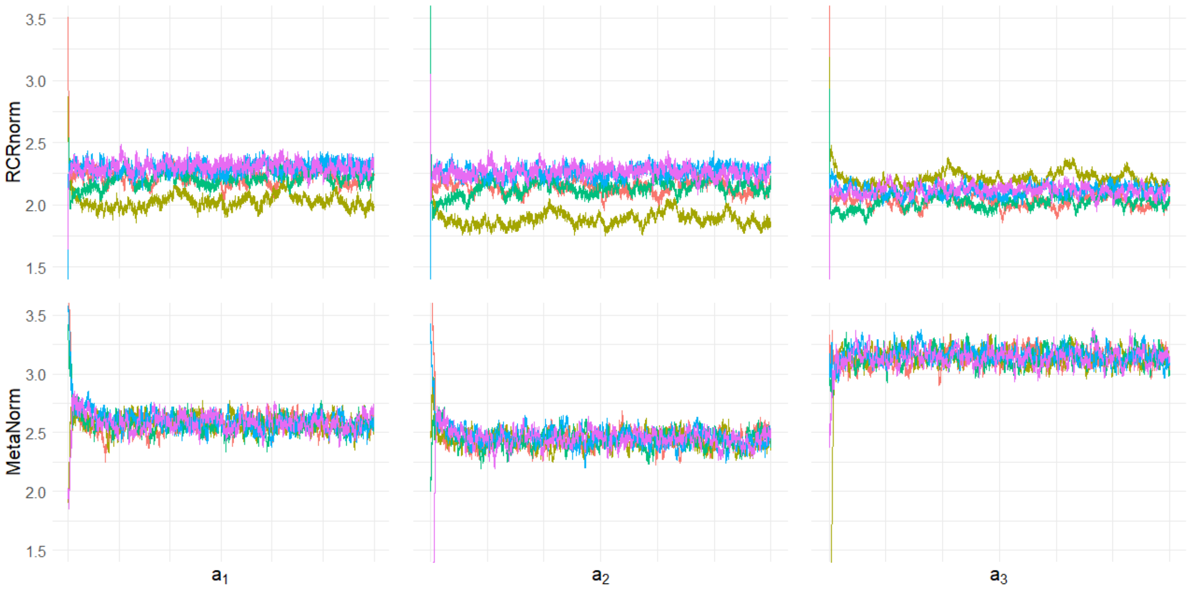

Figure S1: Comparison between RCRnorm and MetaNorm using traceplots of sample-specific intercepts $a_1 - a_3$ (Dataset 13).

S3 Sensitivity Analysis

To confirm our choice of the prior distribution for the covariance matrix $\Sigma^{(k)}$, we conducted a simulation study and evaluated model performance in estimating the key global parameters μ_α and μ_β across various objective priors for the bivariate covariance in the literature, as summarized in Berger and Sun (2008).

[1]Data can be found at https://github.com/RMolania/NanostringNormalization/tree/master/Example%201_LungCancerStudy
[2]Data can be accessed in the RCRnorm R package

Seven simulation settings were included in our study, each of which contains 50 synthetic datasets. For each dataset, we estimated μ_α and μ_β using nine different covariance prior setups: Inverse-Wishart, Scaling, Half-t distribution, Prior J (Jeffery's prior), Prior IJ (Independence Jeffery's prior), Prior $R\rho$, Prior $R\sigma$, Prior $\hat{R}\sigma$, and Prior S. Details about each of these priors can be found in Berger and Sun (2008). Below we list the details of each simulation setting:

- Base: The empirical estimates of the model parameters are used as the truth.
- High Variance (HV): Parameters from the Base setting were used with the magnitudes of σ^2_α and σ^2_β being multiplied by 2.
- Low Variance (LV): Parameters from the Base setting were used with the magnitudes of σ^2_α and σ^2_β being divided by 2.
- Large Meta-Analysis (LMA, i.e., a large number of studies K): Parameters from the Base setting were used. However, the number of studies is doubled.
- Small Meta-Analysis (SMA, i.e., a small number of studies K): Parameters from the Base setting were used. However, the number of studies is halved.
- Large Sample (LS): Parameters from the Base setting were used. However, the number of patients within each study is doubled.
- Small Sample (SS): Parameters from the Base setting were used. However, the number of patients within each study is halved.

To add some more variation into our synthetic datasets, for each setting, we simulated the $\Sigma^{(k)}$'s. Specifically, the variance components of $\Sigma^{(k)}$'s were generated from Inverse-Gamma distributions whose parameters were estimated using moment matching. To simulate the correlation components, we first generated samples from a normal distribution with mean and variance based on observed correlations after Fisher's z-transformation. The variables were then transformed back to the (-1,1) scale. Additionally, we generated the variance for each probe within each study from an Inverse-Gamma distribution where the parameters were estimated using moment matching. Finally, for each study, the number of samples (n_i) was drawn with replacement from the original numbers of patients.

For each covariance setup, we ran an MCMC chain of length 2,500 and burnt in the first half of the chain. To measure the estimation performance of each method, we calculated the MSEs of the estimated posterior means of μ_α and μ_β. The Scaling method took too long to generate posterior sample, so we excluded it from the comparison. The results, scaled by a factor 1000, are summarized in Table S2, which shows that in each simulation setting, the MSE's under the different priors are quite close to each other. Furthermore, there is no clear-cut winner among the priors. For instance, the inverse-Wishart approach has the highest MSE in the base scenario for μ_α but has the lowest MSE in the same scenario for μ_β. These results show that the meta-analysis performance has relatively low sensitivity to the prior choice for $\Sigma^{(k)}$.

	Estimation of μ_α							Estimation of μ_β						
	Base	HV	LV	LMA	SMA	LS	SS	Base	HV	LV	LMA	SMA	LS	SS
IW	2.522	4.392	1.259	1.162	7.941	3.09	4.072	7.741	11.08	4.206	3.347	12.11	6.288	7.489
Half-t	2.456	4.411	1.253	1.171	8.187	3.067	4.097	7.989	10.63	3.898	3.296	12.06	5.712	7.208
Prior J	2.423	4.534	1.289	1.172	8.141	3.053	4.019	7.758	10.29	3.989	3.355	12.27	5.663	7.222
Prior IJ	2.408	4.473	1.278	1.15	8.171	3.087	4.024	7.975	10.01	4.014	3.193	12.41	5.776	7.437
Prior $R\rho$	2.483	4.482	1.316	1.177	8.077	3.039	4.071	7.984	10.36	3.884	3.257	12.69	5.791	6.982
Prior $R\sigma$	2.403	4.44	1.281	1.154	8.085	3.058	4.039	7.876	10.27	4.013	3.268	12.15	5.983	7.489
Prior $\hat{R}\sigma$	2.443	4.447	1.286	1.177	8.113	3.081	4.064	8.029	10.62	4.056	3.277	12.47	5.869	7.429
Prior S	2.396	4.498	1.307	1.175	8.148	3.068	4.075	7.872	10.26	4.022	3.224	11.98	5.865	7.3

Table S2: Analysis of sensitivity to various prior choices of the study-specific covariance matrix Σ^k: 1,000×MSE is reported to evaluate the performance of estimating μ_α and μ_β. LV stands for large variance, SV for small variance, LMA for large meta-analysis, SMA for small meta-analysis, LS for large sample, and SS for small sample.

S4 Additional Enhancements

This section outlines the additional enhancements made to RCRnorm to improve model efficiency, convergence and stability. In addition to the meta-analysis prior, three changes were made to the model:

1. Implementing the constraints $\sum_{p=1}^{P} d_p = 0$, $\sum_{p=1}^{P} d_p X_p^+ = 0$, $\sum_{n=1}^{N} d_n = 0$ in the positive and negative probe equations
2. Updating λ_r, λ_h and ϕ_i with fixed calculations (i.e., no random draws) in each iteration
3. Refurbishing R code with more efficient data structures and procedures to improve computational cost

One other notable update is that MetaNorm does not allow non-randomized starting points. The remainder of this section gives details and justification for the changes listed above.

Constraints on d_p, d_n

In the presence of a high number of local parameters, implementing reasonable constraints can help to stabilize a Gibbs sampler. This is the case with RCRnorm, where random intercepts and slopes (a_i, b_i) are assumed for each sample along with probe-specific effects. In a frequentist analysis of the positive probes, two constraints would be required to achieve a unique solution. Without these constraints, there are an infinite number of solutions, reflecting the fact that predictive power could be distributed in different ways to the probe or sample effects. While this is less severe in an MCMC algorithm, where we rely on thousands of "solutions" (draws) to form a posterior distribution, convergence can sometimes suffer in such a highly complex system such as RCRnorm, especially when the sample size I is not large. This effect can be seen in the trace plots of d_6^+ from the lung cancer FFPE dataset ($I = 28$) used for testing in Jia et al. (2019), shown in Figure S2. The lefthand plot shows a standard run of RCRnorm. While eventually all chains reach a similar range, it takes more than 10,000 draws for this to occur. It is also clear that there is a significant amount of autocorrelation and variability between chains, causing issues of replicability when chains are not long enough. The righthand plot shows 5 chains of RCRnorm with the constraints added. Clearly all chains are quickly converging to the same distribution, with minimal autocorrelation present. These constraints not only help to stabilize d_p^+, but also with other key model parameters including κ_{ir}.

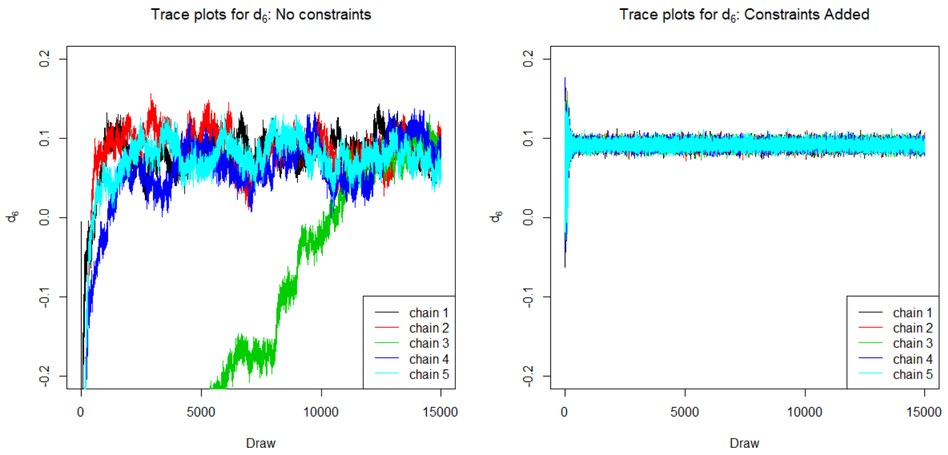

Figure S2: Trace plots of d_6^+ for RCRnorm with and without constraints (right and left, respectively) from the lung cancer FFPE dataset ($I = 28$) used for testing in Jia et al. (2019), showing that the convergence was greatly facilitated by the added constraints.

Updating λ_h, λ_r and ϕ_i

Now we turn our attention to the housekeeping and regular genes. Currently, RCRnorm relies on "safe range" uniform prior distribution for λ_r, λ_h (the mean parameters for $\kappa_{ir}, \kappa_{ih}^*$ respectively) and ϕ_i, which is generated based on empirical estimates of a_i and b_i. Because there are a relatively large number of parameters for the

information provided by the housekeeping and regular gene read counts, the samples for these parameters can oscillate within the pre-defined range, failing to converge to a much narrower distribution as we may expect. Figure S3 shows an example of this behavior using the ϕ_i trace plots from dataset 13 ($I = 8$).

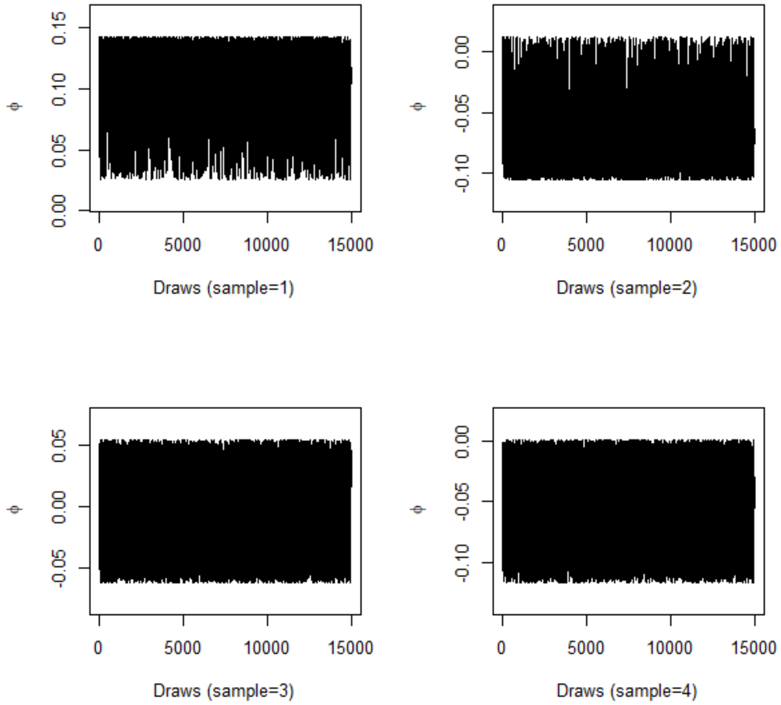

Figure S3: Trace plots for $\phi_1 - \phi_4$ (dataset 13).

Since the model structure is well-justified in Jia et al. (2019), we propose updating these parameters with a fixed calculation to stabilize the ϕ and λ terms, while allowing the κ terms to continue to be updated with its conditional distribution. First, we start by defining

$$\tilde{X}_{ih}^{(t)} = \frac{Y_{ih} - a_i^{(t)}}{b_i^{(t)}} \qquad \tilde{X}_{ir}^{(t)} = \frac{Y_{ir} - a_i^{(t)}}{b_i^{(t)}}$$

which is an estimate of the $\log_{10}$ RNA amount for housekeeping and regular genes given the tth draw of a_i, b_i. Let j index both housekeeping and regular genes such that $j \in (1, ..., H, H+1, ..., H+R = J)$ so that $\tilde{X}_{ij}$ represents $\log_{10}$ RNA from both types of genes. For notational simplicity, we ignore the superscript t here. We propose that the linear fixed-effects model $\tilde{X}_{ij} \sim \phi_i + \lambda_j$ with the constraint $\sum_{i=1}^{I} \phi_i = 0$ be estimated in every loop of the MCMC, with the parameter estimates $\hat{\phi}_i$ and $\hat{\lambda}_j$ used as the draws for ϕ_i, λ_j. While this might seem computationally expensive, the factorial design of the data allows us to take a simple shortcut

$$\hat{\phi}_i = \frac{1}{J}\sum_{j=1}^{J} \tilde{X}_{ij} - \frac{1}{IJ}\sum_{i=1}^{I}\sum_{j=1}^{J} \tilde{X}_{ij} \qquad \hat{\lambda}_j = \frac{1}{I}\sum_{i=1}^{I} \tilde{X}_{ij}.$$

This method allows us to separate the effect of a_i and b_i (driven by the positive probes) so that ϕ_i can focus on the much more subtle sample effects in the housekeeping and regular gene counts. Similarly, this will allow us to obtain stable estimates of λ_h, λ_r which will in-turn stabilize the κ parameters. This will improve model convergence and allow the MetaNorm output to be significantly more precise (as shown in section 3 of Barth et al. (2023)), increasing its value and reliability to researchers.

References

Barth, J., Yang, Y., Xiao, G., and Wang, X. (2023). Metanorm: Incorporating meta-analytic priors into normalization of nanostring ncounter data.

Berger, J. O. and Sun, D. (2008). Objective priors for the bivariate normal model. *The Annals of Statistics*, 36(2):963–982.

Cascione, L., Gasparini, P., Lovat, F., Carasi, S., Pulvirenti, A., Ferro, A., Alder, H., He, G., Vecchione, A., Croce, C. M., Shapiro, C. L., and Huebner, K. (2013). Integrated microrna and mrna signatures associated with survival in triple negative breast cancer. *PloS one; PLoS One*, 8(2):e55910.

Chen, X., Deane, N. G., Lewis, K. B., Li, J., Zhu, J., Washington, M. K., and Beauchamp, R. D. (2016). Comparison of nanostring ncounter.sup.[r] data on ffpe colon cancer samples and affymetrix microarray data on matched frozen tissues. *PloS one*, 11(5).

DeVito, N. C., Sturdivant, M., Thievanthiran, B., Xiao, C., Plebanek, M. P., Salama, A. K. S., Beasley, G. M., Holtzhausen, A., Novotny-Diermayr, V., Strickler, J. H., and Hanks, B. A. (2021). Pharmacological wnt ligand inhibition overcomes key tumor-mediated resistance pathways to anti-pd-1 immunotherapy. *Cell reports (Cambridge); Cell Rep*, 35(5):109071.

Gravemeyer, J., Lange, A., Ritter, C., Spassova, I., Song, L., Picard, D., Remke, M., Horny, K., Sriram, A., Gambichler, T., Schadendorf, D., Hoffmann, D., and Becker, J. C. (2021). Classical and variant merkel cell carcinoma cell lines display different degrees of neuroendocrine differentiation and epithelial-mesenchymal transition. *Journal of investigative dermatology; J Invest Dermatol*, 141(7):1675–1686.e4.

Jia, G., Wang, X., Li, Q., Lu, W., Tang, X., Wistuba, I., and Xie, Y. (2019). Rcrnorm: An integrated system of random-coefficient hierarchical regression models for normalizing nanostring ncounter data. *The annals of applied statistics; Ann Appl Stat*, 13(3):1617–47.

Liu, M. C., Pitcher, B. N., Mardis, E. R., Davies, S. R., Friedman, P. N., Snider, J. E., Vickery, T. L., Reed, J. P., DeSchryver, K., Singh, B., Gradishar, W. J., Perez, E. A., Martino, S., Citron, M. L., Norton, L., Winer, E. P., Hudis, C. A., Carey, L. A., Bernard, P. S., Nielsen, T. O., Perou, C. M., Ellis, M. J., and Barry, W. T. (2016). Pam50 gene signatures and breast cancer prognosis with adjuvant anthracycline- and taxane-based chemotherapy: correlative analysis of c9741 (alliance). *NPJ breast cancer; NPJ Breast Cancer*, 2(1):15023.

Low, Y. S., BlÃ¶cker, C., McPherson, J. R., Tang, S. A., Cheng, Y. Y., Wong, J. Y. S., Chua, C., Lim, T. K. H., Tang, C. L., Chew, M. H., Tan, P., Tan, I. B., Rozen, S. G., and Cheah, P. Y. (2017). A formalin-fixed paraffin-embedded (ffpe)-based prognostic signature to predict metastasis in clinically low risk stage i/ii microsatellite stable colorectal cancer. *Cancer letters; Cancer Lett*, 403:13–20.

Meehan, K., Leslie, C., Lucas, M., Jacques, A., Mirzai, B., Lim, J., Bulsara, M., Khan, Y., Wong, N. C., Solomon, B., Sader, C., Friedland, P., Arnau, G. M., Semple, T., and Lim, A. M. (2020). Characterization of the immune profile of oral tongue squamous cell carcinomas with advancing disease. *Cancer medicine (Malden, MA); Cancer Med*, 9(13):4791–4807.

Molania, R., Gagnon-Bartsch, J., Dobrovic, A., and Speed, T. P. (2019). A new normalization for nanostring ncounter gene expression data. *Nucleic acids research; Nucleic Acids Res*, 47(12):6073–6083.

Nielsen, P. R., Eriksen, J. O., Lindahl, L. M., Wehkamp, U., Andersen, G. R., Bzorek, M., Litman, T., ødum, N., and Gjerdrum, L. M. R. (2019). 029 - a diagnostic two gene classifier in patients with mycosis fungoides: a retrospective multicenter study. *European journal of cancer (1990)*, 119:S11.

Patel, A., GarcÃa-Closas, M., Olshan, A. F., Perou, C. M., Troester, M. A., Love, M. I., and Bhattacharya, A. (2022). Gene-level germline contributions to clinical risk of recurrence scores in black and white patients with breast cancer. *Cancer research (Chicago, Ill.); Cancer Res*, 82(1):25–35.

Pu, M., Messer, K., Davies, S. R., Vickery, T. L., Pittman, E., Parker, B. A., Ellis, M. J., Flatt, S. W., Marinac, C. R., Nelson, S. H., Mardis, E. R., Pierce, J. P., and Natarajan, L. (2019). Research-based pam50 signature and long-term breast cancer survival. *Breast cancer research and treatment; Breast Cancer Res Treat*, 179(1):197–206.
